# No evidence that attentional bias towards pain-related words is associated with verbally induced nocebo hyperalgesia: a dot-probe study

**DOI:** 10.1097/PR9.0000000000000921

**Published:** 2021-04-06

**Authors:** Matthew James Coleshill, Louise Sharpe, Ben Colagiuri

**Affiliations:** a School of Psychology, The University of Sydney, Sydney, Australia; b St. Vincent's Clinical School, UNSW Medicine, UNSW Sydney, Sydney, Australia; c Department of Clinical Pharmacology and Toxicology, St Vincent's Hospital, Sydney, Australia

**Keywords:** Nocebo effect, Nocebo hyperalgesia, Attentional bias, Anxiety

## Abstract

**Introduction:**

Placebo and nocebo effects in pain are well documented. One leading explanation is that instructions indicating that pain will either increase or decrease after receipt of a treatment give rise to expectations for increased or decreased pain. However, the psychological mechanisms through which expectations affect pain perception are not well understood. One possibility is that the expectation of increased pain leads to anticipatory anxiety, which in turn increases attention towards painful sensations.

**Objectives:**

The aim of this study was to test the hypothesis that attention mediates nocebo hyperalgesia. This was done by measuring attentional bias towards pain using a dot-probe task both before and after a nocebo manipulation.

**Methods:**

Ninety-six healthy volunteers were randomized to receive one of the following: (1) an inert nasal spray with placebo instructions, (2) an inert nasal spray with nocebo instructions, or (3) no treatment. Participants completed measures of expectations, anxiety, and attention bias (dot-probe paradigm) both before and after randomization.

**Results:**

Results showed that the nocebo instructions induced expectations for increased pain and resulted in nocebo hyperalgesia. Conversely, the placebo instruction failed to induce expectations for decreases in pain and did not demonstrate any placebo analgesia. Furthermore, despite the significant expectancies for pain and subsequent nocebo hyperalgesia, there were no differences between the nocebo group and either the placebo or no-treatment group for anxiety or attentional bias.

**Conclusion:**

The results are consistent with the expectancy model of placebo and nocebo effects. That is, changes in expectations seemed to be necessary to induce a placebo or nocebo effect. However, there was no evidence that anxiety or attention bias towards pain-related stimuli was necessary to achieve nocebo hyperalgesia.

## Abstract 2

Supplemental Digital Content is Available in the Text.

This study examined attention as a mechanism of nocebo hyperalgesia. No association was observed between attentional bias towards pain-related words and nocebo hyperalgesia.

## 1. Introduction

Placebo analgesia and nocebo hyperalgesia are well-established phenomena both experimentally and clinically. Although expectations of decreased or increased pain seem to drive both placebo analgesia and nocebo hyperalgesia, there is evidence that they differ mechanistically. For example, anxiety has been suggested to be a central component of nocebo hyperalgesia,^2,7^ whereas there is mixed evidence that reductions in anxiety may facilitate placebo analgesia.^20,23^

Despite the inclusion of anxiety in models of nocebo hyperalgesia, behavioural findings are mixed, with a number of studies finding no association between nocebo manipulations and anxiety,^18,25,34^ whereas others do observe such relationships.^6,9,16^ One explanation for these inconsistencies may be how anxiety contributes to nocebo hyperalgesia. Colloca and Benedetti^7^ proposed that nocebo treatments elicit anticipatory anxiety that causes increased attention to pain, which in turn enhances pain experience as a result of hypervigilance. This account may explain the inconsistencies in the literature as it may be attentional processes that produce hyperalgesia, rather than anxiety per se.

Despite a rich history of research on attention and pain, no research has empirically tested whether attention mediates nocebo hyperalgesia. To test this hypothesis, we incorporated an established and widely used measure of attentional bias to pain, the dot-probe task,^21^ in a study comparing placebo analgesia and nocebo hyperalgesia. The dot-probe task was chosen because of its established use as a tool for examining attentional biases, as well as its association with pain status.^31^ Participants were randomised to receive either placebo instruction, nocebo instruction, or no treatment. A dot-probe task was completed at both baseline and after treatment instruction. If heightened attention to pain underlies nocebo hyperalgesia specifically, then the nocebo group should demonstrate a significant attentional bias to pain, which mediates any nocebo hyperalgesia they experience, with no attentional effects observed in the placebo group.

## 2. Methods

### 2.1. Design

The study used a 3 (instruction: placebo instructions, nocebo instructions, or no instructions) × 2 (phase: pretreatment or posttreatment) mixed design. Instruction was manipulated between subjects, whereas phase was manipulated within subjects. Attentional bias towards pain was assessed through a dot-probe task. The primary measures were pain threshold and tolerance. Secondary measures were attentional bias, state and trait anxiety, fear of pain, and expectations of pain and treatment efficacy.

### 2.2. Participants and randomisation

Ninety-six (71 female) undergraduates with an average age of 19.33 (SD = 2.95) participated in the study. Exclusion criteria for the experiment were the current use of any prescribed or unprescribed analgesics or the experience of pain at the time of the study. Participants were assigned to receive placebo instructions, nocebo instructions, or no-treatment instructions using block randomisation with block sizes of 6. Randomisation was concealed until after baseline assessment. The randomisation sequence was developed by an independent researcher. The experimental procedure was approved by the Human Research Ethics Committee of the University of Sydney.

### 2.3. Dot-probe task

Attentional bias was assessed using the dot-probe paradigm,^4,21^ which was administered on a laptop computer with a 35.5-cm (diagonal) 1366 × 768 resolution monitor. A yellow fixation point “.” was presented in the centre of the monitor that displayed a blue background for 500 ms. In critical trials, a pair of words were presented, one pain related and one neutral. For noncritical trials, neutral/neutral stimuli were presented. Word stimuli were yellow, lower case, 5 mm in size, and tahoma typeface. The words appeared 2 cm above or below the central point. The word pair remained on the monitor for either 500 or 1250 ms, depending on the trial, before they were replaced with a “p” or a “q,” which was presented in one of the word's locations. Participants were instructed to indicate whether the p or q was presented by using the “p” or “q” key on a keyboard as quickly as possible. The trial terminated on the participant's response or after 1500 ms had elapsed. The intertrial interval was 500 ms.

Five practice trials using neutral/neutral words were presented at the beginning. Three sets of stimuli were used: sensory/neutral, affective/neutral,^14^ and neutral/neutral pairs^13^ (Table 1). Two presentation times of 500 and 1250 ms were included to determine attention during the initial fixation (500 ms) or subsequent attention (1250 ms).^12^ Two presentation times were used to examine whether any attentional bias was due to speeded responses to congruent trials, indicative of hypervigilance, or due to slowed responses to incongruent trials, indicating difficulty disengaging from the pain-related stimuli.^19^

**Table 1:** Word pairs used in the dot-probe task.

Sensory/neutral	Affective/neutral	Neutral/neutral
Flickering/neutral	Vicious/lessons	Bedroom/surface
Throbbing/sailboat	Annoying/chivalry	Bleach/cooker
Shooting/drinking	Miserable/undertake	Brushing/decorate
Boring/swivel	Troublesome/restraining	Container/staircase
Drilling/whirling	Unbearable/metabolite	Doorknob/bathroom
Sharp/items	Cruel/drums	Furniture/magazines
Burning/moment	Tiring/cotton	Housework/lightbulb
Stiff/skirt	Exhausting/blackberry	Rack/plug
Tugging/refresh	Punishing/advocates	Towels/bedspread
Pinching/postmark	Discouraging/subcommittee	Vase/tidy

Each set contained 10 unique word pairings, presented in one of 4 combinations, which varied depending on the site of presentation of the target or probe in the upper or lower half of the screen. For the pain/neutral trials, this resulted in 2 types of trials. In congruent trials, the target and probe were presented in same position (either the upper or lower half of the screen). In incongruent trials, the words were presented in different locations. An attentional bias towards pain-related stimuli is inferred when participants respond more quickly to congruent than incongruent trials. In this study, each set of 30 word pairs were presented twice, once with a presentation time of 500 ms and the other with a presentation time of 1250 ms, in blocks, the order of which was randomized. There was a total of 240 trials. The task was given twice, before and after the instruction manipulation to determine changes in attentional biases. There were 2 alternate versions that were presented in a counterbalanced order to control for order effects.

### 2.4. Calculation of attentional bias

Indices of attentional bias were calculated using the following formula, in which t = target word; p = probe; u = upper location; and l = lower location:

([tupl−tlpl]+[tlpu−tupu])/2

### 2.5. Pain assessment

A contact thermode (PATHWAY CHEPS, Medoc Ltd, Ramat Yishai, Israel) was used to deliver thermal nociceptive stimuli. Pain threshold and tolerance were measured using a series of 6 stimuli with a 20-second interstimulus interval.^24^ All stimuli began at 32°C, increased at a rate of 0.5°C/second, and decreased at a rate of 10°C/second. The first 3 stimuli assessed pain threshold. Using a handheld button the participant indicated the point at which each stimulus became painful, on which the trial ended. The average of these trials was the pain threshold. The second 3 stimuli assessed pain tolerance in which the participant used the handheld button to indicate when they could no longer tolerate the stimuli. The average of these trials was taken as pain tolerance. Safety parameters were used so that the stimuli could never exceed 51.5°C. Participants who reached this parameter were scored as 51.5°C.

### 2.6. Placebo and nocebo instructions

The placebo and nocebo treatments took the form of 5 mL of saline with 0.06% ethanol solution nasal sprays. Participants self-administered 4 doses, 2 in each nostril. In the placebo group, the nasal spray was labelled “Lidocaine Nasal Solution,” and participants were informed that this was a fast-acting anaesthetic. In the nocebo group, the nasal spray was labelled “Naloxone Hydrochloride Nasal Solution,” and participants were informed that this was a treatment that would increase their pain. The specific wording of the instruction conditions is provided in the Supplementary Materials (available at http://links.lww.com/PR9/A102). In the no-treatment condition, participants were simply informed that they would not receive treatment.

### 2.7. Questionnaire assessments

Anxiety was measured using the State-Trait Anxiety Inventory (STAI),^28^ which assesses both state (STAI-S) and trait (STAI-T) anxiety. Fear of pain was assessed using the FPQ-III.^22^ Finally, a questionnaire was administered that assessed both expectations of treatment efficacy and expectations of pain (see Supplementary Materials, available at http://links.lww.com/PR9/A102).

### 2.8. Procedure

Participants were told the study examined how pharmacological treatments alter subjective and psychophysiological outcomes related to pain. Participants completed the dot-probe task to obtain baseline measures of attentional bias; after which baseline pain threshold and tolerance were measured. After this, participants completed the STAI-S. Participants were then randomised and received either the placebo instructions, nocebo instructions, or no instructions. During a waiting period of 10 minutes, participants completed the remaining questionnaires. After this, participants completed a second dot-probe task and the STAI-S, before pain threshold and tolerance were once again measured. All participants were subsequently debriefed.

### 2.9. Data analysis

To test for between group differences, baseline measures were analysed using one-way analyses of variance (ANOVAs) with instruction (placebo, nocebo, or no treatment) as the independent variable. Internal consistency for the STAI was examined using the Cronbach alpha. Expectations of treatment efficacy and posttreatment pain were examined using one-way ANOVAs with instruction as the independent variable, followed by pairwise comparisons adjusted using the Bonferroni correction. Changes in pain and anxiety between the pretreatment and posttreatment phases were analysed using 3 (instruction) × 2 (phase: pretreatment or posttreatment) mixed model ANOVAs. Where significant, pairwise comparisons on the difference scores were conducted and adjusted using the Bonferroni correction. Cohen *d* was calculated using the following formula:

$$d=\text{M}1-\text{M}2/\sqrt{{\text{MSE}}_{\text{within}}}$$

Attentional bias data were analysed using 3 (instruction) × 2 (stimuli: affective or sensory) × 2 (phase) mixed model ANOVAs, with instruction as the between subjects factor. Separate mixed model ANOVAs were conducted for the 500 and 1250 ms presentation times. Correlational analyses between psychological characteristics and pain outcomes were performed using bivariate correlations. Data were analysed using IBM SPSS Statistics for Windows, version 26 (IBM Corp, Armonk, NY).

## 3. Results

Data from 8 participants were removed because of hypersensitivity to thermal stimuli (pain threshold <40°C). This criterion was based on the literature for allodynia, where a 40°C stimulus is often used as a warm stimulus and the presence of pain in response to such a stimulus is considered evidence of allodynia.^5^ Sensitivity analyses indicated no difference in results when these participants were removed. Data from 2 further participants were removed because of having a greater than 50% error rate on the dot-probe task. Hence, data from 86 participants were analysed, 29 in placebo instruction, 31 in nocebo instruction, and 26 in no treatment. Ceiling values for pain tolerance (51.5°C) were reached for 14 participants pretreatment and 15 participants posttreatment. Sensitivity analyses were conducted by excluding all participants who reached the ceiling value of 51.5°C. The pattern of results was unchanged.

### 3.1. Baseline data

One-way ANOVAs revealed no significant between-group differences on any baseline variables: all *F’s*(2, 85) ≤ 2.230, all *p’s* ≥ 0.114 (Table 2). Tests of internal consistency observed good internal consistency for both the state (α = 0.868) and trait (α = 0.904) components of the STAI.

**Table 2:** Mean baseline data with SD for pain threshold and tolerance as well as measures of trait anxiety and fear of pain as a function of instruction group.

	Gender	Age	Pain threshold	Pain tolerance	Trait anxiety	Fear of pain
Placebo	8M/21F	19.38 (2.67)	44.72 (2.01)	48.53 (2.20)	44.34 (11.39)	16.91 (3.14)
Nocebo	11M/20F	19.03 (2.52)	44.90 (1.90)	48.79 (2.05)	41.81 (8.42)	16.67 (3.00)
Control	6M/19F	20.00 (4.04)	44.94 (2.08)	48.52 (2.00)	39.23 (6.08)	19.65 (3.86)

### 3.2. Posttreatment analyses

#### 3.2.1. Expectations

The one-way ANOVA found a significant between-groups effect on expectations of treatment efficacy: *F*(2, 85) = 29.7, *P* < 0.001. Pairwise comparisons indicated that the placebo (M = 5.31, SD = 2.02, 95% confidence interval [CI]: 4.54-6.08, *d* = 1.66) and nocebo groups (M = 5.84, SD = 1.90, 95% CI: 5.14-6.54, *d* = 1.92) had greater expectations that the treatment would change their pain perception than the no-treatment group (M = 1.85, SD = 2.34, 95% CI: 0.90-2.79, *p*s < 0.001). No difference was observed between the 2 instruction groups: *P* = 0.986, *d* = 0.25.

The second one-way ANOVA examining expectations of pain also found group differences (*F*(2, 85) = 7.825, *P* = 0.001). Pairwise comparisons confirmed expectations of pain were significantly higher in the nocebo group (M = 7.06, SD = 1.44, 95% CI: 6.54-7.59) compared with those of both the placebo (M = 5.48, SD = 1.90, *P* = 0.002, 95% CI: 4.76-6.21, *d* = 0.93) and no-treatment groups (M = 5.65, SD = 1.74, *P* = 0.007, 95% CI: 4.95-6.36, *d* = 0.83). No significant difference in expectations of pain was observed between the placebo and no-treatment groups: *P* > 0.999, *d* = 0.1.

#### 3.2.2. Pain threshold and tolerance

**Figure 1.**
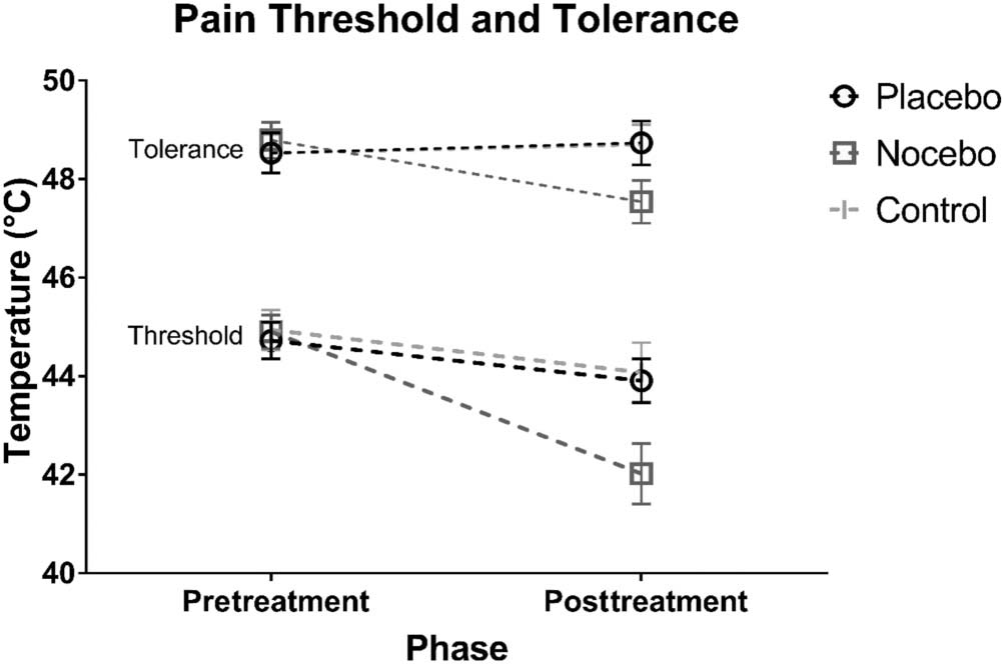
Graph depicting average pain threshold and tolerance (°C) with standard error bars as a function of phase and instruction conditions.

The 3 (instruction) × 2 (phase) mixed model ANOVA for pain threshold (Fig. 1) revealed a significant main effect of phase: *F*(1, 83) = 29.904, *P* < 0.001, ${\text{η}}_{\text{p}}^{2}$ = 0.265, as well as a significant interaction between instruction and phase: *F*(2, 83) = 6.260, *P* = 0.003, ${\text{η}}_{\text{p}}^{2}$ = 0.131. The main effect of instruction was not statistically significant: *F*(2, 83) = 1.896, *P* = 0.157. Pairwise comparisons found that the change in pain threshold between the pretreatment and posttreatment phases was significantly greater during the nocebo manipulation (M = −2.89, SD = 2.91, 95% CI: −3.95 to −1.81) compared with that in the placebo (M = −0.82, SD = 2.37, *P* = 0.008, 95% CI: −1.72 to 0.08, *d* = 0.80) and no-treatment conditions (M = −0.86, SD = 2.35, *P* = 0.012, 95% CI: −1.81 to 0.09, *d* = 0.79). No difference was observed between the placebo manipulation and the no-treatment control (*P* > 0.999, *d* = 0.02).

Analysis of the pain tolerance data (Fig. 1) using a similar mixed ANOVA also found a nonsignificant main effect of phase (*F*(1, 83) = 3.82, *P* = 0.052, ${\text{η}}_{\text{p}}^{2}$ = 0.045), but a significant interaction between instruction and phase (*F*(2, 83) = 11.051, *P* < 0.001, ${\text{η}}_{\text{p}}^{2}$ = 0.210). The main effect of instruction was not significant (*F*(2, 83) = 0.468, *P* = 0.628). Pairwise comparisons found that the difference in pain tolerance between the pretreatment and posttreatment phases was significantly greater during the nocebo manipulation (M = −1.25, SD = 1.87, 95% CI: −1.93 to −0.56) compared with that in the placebo (M = 0.20, SD = 1.03, *P* < 0.001, 95% CI: −0.19 to 0.60, *d* = 1.07) and no-treatment conditions (M = 0.17, SD = 0.89, *P* = 0.001, 95% CI: −0.19 to 0.53, *d* = 1.04), whereas no difference was observed between the placebo manipulation and the no-treatment control: *P* > 0.999, *d* = 0.02.

#### 3.2.3. State anxiety

State anxiety (Table 3), examined using a 3 (instruction) × 2 (phase) mixed model ANOVA, found no significant main effects of phase (*F*(1, 83) = 0.002, *P* = 0.966), instruction (*F*(2, 83) = 0.316, *P* = 0.730), nor any interaction between phase and instruction (*F*(2, 83) = 1.413, *P* = 0.249). This indicated that state anxiety did not differ between the instruction groups nor within instruction group between the pretreatment and posttreatment phases of the experiment.

**Table 3:** Mean state anxiety with SD as a function of instruction condition and phase.

	State anxiety	
Pre		Post
Placebo	34.72 (9.22)	33.17 (8.57)
Nocebo	32.06 (5.74)	33.03 (6.05)
Control	32.81 (8.21)	33.31 (6.86)

#### 3.2.4. Attentional bias towards pain

Measures of attentional bias towards pain (Table 4) were analysed using 2 (phase) × 2 (stimuli) × 3 (instruction) mixed model ANOVAs. The first of these examined data from trials using 500 ms stimulus presentation, finding no significant main effects of phase (*F*(1, 83) = 0.547, *P* = 0.461), instruction (*F*(2, 83) = 1.097, *P* = 0.339), or stimulus (*F*(1, 83) = 0.045, *P* = 0.833), nor interaction between phase and instruction (*F*(2, 83) = 0.202, *P* = 0.817), phase and stimulus (*F*(1, 83) = 0.514, *P* = 0.475), stimulus and instruction (*F*(2, 83) = 1.239, *P* = 0.295), or phase, stimulus, and instruction (*F*(2, 83) = 0.679, *P* = 0.510).

**Table 4:** Mean attentional bias with SD as a function of stimulus presentation, instruction condition, and phase.

	500 ms				1250 ms			
	Affective		Sensory		Affective		Sensory	
	Pre	Post	Pre	Post	Pre	Post	Pre	Post
Placebo	−7.42 (49.19)	−16.22 (58.96)	−6.98 (46.46)	3.39 (73.74)	−9.60 (61.54)	2.80 (54.59)	8.78 (46.37)	0.43 (53.79)
Nocebo	−3.93 (52.90)	−4.57 (54.29)	6.60 (53.17)	−4.17 (50.83)	−9.92 (81.25)	0.75 (72.87)	13.98 (46.64)	2.56 (39.42)
Control	16.90 (51.75)	0.74 (40.97)	−2.73 (53.27)	−3.22 (36.18)	−0.99 (53.23)	0.69 (38.23)	9.39 (42.82)	−0.60 (40.90)

For trials using 1250 ms stimulus presentation, once again finding no significant main effects of phase (*F*(1, 83) = 0.022, *P* = 0.883), instruction (*F*(2, 83) = 0.026, *P* = 0.975), or stimulus (*F*(1, 83) = 0.1.813, *P* = 0.182), nor interaction between phase and instruction *F*(2, 83) = 0.097, *P* = 0.908), phase and stimulus (*F*(1, 83) = 2.371, *P* = 0.127), stimulus and instruction *F*(2, 83) = 0.148, *P* = 0.863), or phase, stimulus, and instruction (*F*(2, 83) = 0.074, *P* = 0.929).

#### 3.2.5. Correlational analysis

As only the nocebo instruction group exhibited any significant changes in pain perception, correlational analyses were only performed on this group. This analysis revealed a significant correlation between changes in pain threshold between the pretreatment and posttreatment phases with attentional bias towards affective words presented at 500 ms (*r*(29) = 0.42, *P* = 0.019). No other significant correlations were observed (Table 5). A complete correlation matrix is available in the Supplementary Materials (available at http://links.lww.com/PR9/A102).

**Table 5:** Correlation matrix depicting pain threshold and tolerance in the posttreatment phase and differences between the pretreatment and posttreatment phases during nocebo instructions with measures of state and trait anxiety, fear of pain, expectations of treatment efficacy and pain, as well as measures of attentional bias.

	Threshold	Threshold pre–post	Tolerance	Tolerance pre–post
State anxiety	0.291	0.052	0.337	−0.054
State anxiety pre–post	0.209	0.100	0.059	−0.112
Expectancy treatment	−0.066	−0.091	−0.008	0.017
Expectancy pain	−0.041	−0.272	0.334	0.121
Trait anxiety	0.042	−0.140	0.006	−0.201
Fear of pain	0.012	0.090	−0.117	0.065
Fear of pain—medical	−0.126	−0.064	−0.160	−0.019
Fear of pain—severe	−0.042	0.098	−0.173	0.014
Fear of pain—minor	0.169	0.200	−0.012	0.156
Attentional bias affective 500 ms	0.348	0.418*	0.098	0.301
Attentional bias sensory 500 ms	0.030	0.106	−0.053	−0.064
Attentional bias affective 1250 ms	−0.225	−0.224	0.096	−0.083
Attentional bias sensory 1250 ms	0.276	0.131	0.323	0.332

* Significant correlations are marked with a (*P* < 0.05).

## 4. Discussion

This study examined whether attentional bias towards pain-related information, as assessed through a dot-probe task, was associated with changes in pain brought about by either placebo or nocebo instructions. A significant nocebo effect was observed on both pain threshold and tolerance relative to no treatment. No changes, however, were observed in anxiety or attentional bias after the nocebo instruction. The only evidence suggestive of a link between attentional bias and nocebo hyperalgesia was a single significant correlation between changes in pain threshold (but not tolerance) and attentional bias towards affective words presented at 500 ms, with no significant correlations between any other attentional bias index and either pain threshold or tolerance. These findings suggest that attentional bias towards pain-related information could not explain the subsequent nocebo hyperalgesia. Instead, the primary factor that seemed to drive the observed nocebo hyperalgesia was expectations, as demonstrated by heightened expectations of pain experience brought about by the nocebo instructions. By contrast, no effect of the placebo manipulation was observed on pain threshold or tolerance relative to no treatment, indicating no evidence of placebo analgesia.

Colloca and Bendetti's^7^ model of nocebo hyperalgesia proposed that the expectation of exacerbated pain increases anxiety and subsequent attention to pain. According to their model, attentional bias was suggested to mediate the effect of nocebo-induced anxiety on pain. Our results, however, disconfirmed this prediction in 2 ways. First, the finding that nocebo instructions increased expectations and subsequent pain report but did not produce consistent changes in attentional processes. This suggests that attentional processes (at least those measured by the dot-probe task) are not necessary to produce nocebo hyperalgesia. Interpretation of this finding, however, is complicated by the second inconsistency between the model and the present findings—the failure of the nocebo instruction to have an observable effect on anxiety. This lack of relationship is unlikely to be due to the strength of the nocebo effect in this study, which observed substantial reductions in pain threshold (*d* = 0.79) and pain tolerance (*d* = 1.04) relative to no treatment. The observation of a robust nocebo effect in the absence of changes in anxiety suggests that such a model cannot entirely account for the psychological mechanisms of the nocebo effect because we observed nocebo hyperalgesia without any apparent increases in anxiety.

Despite the theoretical importance assigned to the role of anxiety in nocebo hyperalgesia,^2,7^ only one other study has examined changes in anxiety prenocebo and postnocebo manipulation. In that study, higher state anxiety was observed in the nocebo group compared with other treatment conditions, although increases in pretreatment and posttreatment were not analysed.^16^ Although these findings are difficult to reconcile, it should be noted that other behavioural evidence from the literature is also mixed,^17^ with some studies observing correlations between nocebo hyperalgesia and state anxiety^9^ and others not.^11,18,34^ Taken together, such findings may suggest that generalised anxiety after nocebo instructions is a by-product of the manipulation, rather than a mediating component.^17^ Alternatively, measures of state anxiety, such as the STAI, may not be sufficiently sensitive to reliably detect the changes in anxiety-inducing nocebo hyperalgesia.

Instead, the primary factor in the nocebo effect in the current study was expectation. The general theories of placebo and nocebo effects indicate changes are produced through expectations.^8^ In this study, we found that the nocebo instructions did elicit expectations of more pain, whereas the placebo instruction did not produce expectations of less pain. Thus, these results support the role of expectations, in that where the instruction changed expectations there was a subsequent change in pain outcomes, while where the instruction failed to influence expectations, no effect was found. Nonetheless, if expectations were the primary mechanisms, correlations between expectancy and changes in pain would be expected to be large, which was not the case in this study. It should be noted, however, that the correlational analyses were likely underpowered as the sample size in each group was relatively small. Nevertheless, our results are consistent with the hypothesis that nocebo instruction elicited heightened expectancies of pain, whereas placebo instruction failed to elicit expectations of benefit.

If changing expectations of pain is necessary to induce both placebo and nocebo effects, then the failure of the placebo effect in this study is consistent with theory, given that the placebo manipulation was unsuccessful in changing expectations. Importantly, our procedure for both treatment pretext^15^ and method of administration^24^ was consistent with previous research where placebo effects have been observed. The most likely explanation of the lack of an effect of the placebo manipulation is that experimentally induced placebo analgesia seems much less responsive to verbal suggestions compared with nocebo hyperalgesia.^10^ Corroborating this, a meta-analysis found placebo analgesia induced by instructions alone was substantially weaker than when verbal instructions were paired with surreptitious conditioning.^33^ As such, the available evidence is consistent with a higher likelihood of inducing nocebo hyperalgesia through instructions alone compared with placebo analgesia.

There are some limitations to this study. First, it could be the case that the dot-probe task was not sufficiently sensitive to detect any attentional bias induced by the nocebo manipulation. Although there is evidence that threat manipulations regarding pain do influence attentional biases towards pain-related stimuli,^3,26^ it is worth highlighting that the threat manipulation in these studies specifically aimed to increase the threat of pain. Indeed, in those studies, manipulation checks confirmed that participants came to expect more harm associated with pain and were more worried about the pain.^3^ Thus, it may be that increasing the threat or anxiety associated with pain is necessary to find differences on attentional processes. Second, there is also ongoing debate regarding the best method of assessing attentional bias towards pain. Tasks, such as the dot-probe, that rely on assessing attentional bias towards stimuli (such as words) that represent pain have been challenged because if attention is shown to be a putative mechanism in the perception of pain, it is likely that it is attention to painful sensations not representations of them.^32^ In this study, we used previously validated words rather than words developed specifically for the thermal pain task, which may have compounded this problem. Third, the dot-probe, when based on reaction times, provides only a snapshot of attention at the time when the probe appears. As such, it is perhaps unsurprising that attention biases based on reactions times have been found to be unreliable.^13^ For that reason, recent studies have relied on eye tracking to provide a more direct assessment of attention, and future research should use more direct methods of assessment.^27,30^ Furthermore, it has been highlighted that attentional biases are dynamic processes and are likely context dependent.^32^ Attentional bias towards pain may have only been present during the pain task itself, leading to an absence of attentional bias during the separate dot-probe task. Further research should attempt to better integrate the attentional bias and pain tasks to clarify this question. Fourth, the sample size used in the study was based on conventions to observe either the placebo or nocebo effects. Although the sample size is similar to other dot-probe studies,^1,29^ it remains possible that a lack of power could have led to the failure to detect differences in attentional bias. Future studies could use the current data for a priori power calculations. Finally, because we only assessed verbally induced nocebo hyperalgesia, it remains possible that attentional bias (and anxiety) could mediate other types of nocebo hyperalgesia (eg, conditioned and socially induced).

In conclusion, despite clear evidence of nocebo hyperalgesia, this study found no evidence that anxiety or attentional biases were necessary to induce nocebo hyperalgesia. This challenges common conceptions that anxiety may be a key mechanism of nocebo hyperalgesia, whereby anxiety has been proposed to induce attentional biases towards pain that in turn exacerbate pain. Rather, our results are consistent with the expectancy model of nocebo hyperalgesia, in which changes in expectation are associated with hyperalgesia.^8^ Nonetheless, the precise psychological mechanisms through which expectations lead to changes in pain remain unclear. In addition, there was no effect of a change in expected efficacy of a treatment during placebo instruction on what should be an analgesic effect. Future research should explore other measures of attentional bias using eye tracking and whether conditioning or social nocebo manipulations affect attentional bias differentially to purely instructional manipulations, as well as when a placebo analgesic effect is evident.

## Disclosures

The authors have no conflicts of interest to declare.

### Supplementary Material

SUPPLEMENTARY MATERIAL

## Appendix A. Supplemental digital content

Supplemental digital content associated with this article can be found online at http://links.lww.com/PR9/A102.
