# Supplementary material for: No evidence that attentional bias towards pain-related words is associated with verbally induced nocebo hyperalgesia: a dot-probe study
Source: Pain Rep. 2021 Apr 6;6(1):e921. doi: 10.1097/PR9.0000000000000921 (PMC8108596; doi:10.1097/PR9.0000000000000921)
Supplement: SUPPLEMENTARY MATERIAL [file painreports-6-e921-s001.docx]

Supplementary Material 1. Specific wording employed in instruction conditions.

Placebo instruction condition:

*‘You have been allocated to the analgesic condition, where you will receive a pain-relieving anaesthetic called Lidocaine. Lidocaine is a fast-acting anaesthetic with a relatively short-lasting effect which acts by numbing the receptors on your skin responsible for transferring pain-related information. In this way, less information about a source of pain is transferred from your skin to your brain, leading to a reduced sensation of pain while the drug is in effect.’*

Nocebo instruction condition:

*‘You have been allocated to the hyperalgesic condition, where you will receive a pain-increasing drug called Naloxone. Now, your experience of pain is the product of the sensory information from a potentially damaging source being interpreted by the brain, with this interpretation being able to reduce or increase the perception of pain. In this way, your brain has inbuilt systems for both increasing and decreasing the experience of pain based on the situation at hand, with the balance between these systems being your resulting experience. Naloxone is an opioid antagonist, which means that it interferes with your brain’s inbuilt pain-relieving system, known as the endogenous opioid system. By temporally interfering with this system, your perception of pain should increase for a short period.’*

Supplementary Material 2. Questionnaires employed to assess expectations of treatment efficacy and expectations of pain. The questionnaires varied between instruction conditions and examined how effective the participant believed their respective treatment (placebo, nocebo, no treatment) would be at altering (decreasing, increasing, changing) their pain sensitivity, and how much pain they expected to experience. These questions were rated on an 11-point numerical rating scale ranging from ‘not at all’ to ‘very effective’ and ‘no pain’ to ‘very painful’. The questionnaire had two foil questions relating to how comfortable the participant was and their current heart rate.

Questionnaire 1. Placebo instruction condition.

1. How comfortable do you feel right now?

| 0 | 1 | 2 | 3 | 4 | 5 | 6 | 7 | 8 | 9 | 10 |
| --- | --- | --- | --- | --- | --- | --- | --- | --- | --- | --- |
| Not at all |  | |  |  |  |  |  |  | Very comfortable | |

1. How is your heart rate right now?

| 0 | 1 | 2 | 3 | 4 | 5 | 6 | 7 | 8 | 9 | 10 |
| --- | --- | --- | --- | --- | --- | --- | --- | --- | --- | --- |
| Completely  normal | | |  |  |  |  |  |  | Very  affected | |

1. How effective do you think the lidocaine nasal spray will be for decreasing your pain sensitivity?

| 0 | 1 | 2 | 3 | 4 | 5 | 6 | 7 | 8 | 9 | 10 |
| --- | --- | --- | --- | --- | --- | --- | --- | --- | --- | --- |
| Not at all |  | |  |  |  |  |  |  | Very effective | |

1. How much pain do you expect on the pain test?

| 0 | 1 | 2 | 3 | 4 | 5 | 6 | 7 | 8 | 9 | 10 |
| --- | --- | --- | --- | --- | --- | --- | --- | --- | --- | --- |
| No pain |  | |  |  |  |  |  |  | Very  painful | |

Questionnaire 2. Nocebo instruction condition.

1. How comfortable do you feel right now?

| 0 | 1 | 2 | 3 | 4 | 5 | 6 | 7 | 8 | 9 | 10 |
| --- | --- | --- | --- | --- | --- | --- | --- | --- | --- | --- |
| Not at all |  | |  |  |  |  |  |  | Very comfortable | |

1. How is your heart rate right now?

| 0 | 1 | 2 | 3 | 4 | 5 | 6 | 7 | 8 | 9 | 10 |
| --- | --- | --- | --- | --- | --- | --- | --- | --- | --- | --- |
| Completely  normal | | |  |  |  |  |  |  | Very  affected | |

1. How effective do you think the naloxone hydrochloride nasal spray will be for increasing your pain sensitivity?

| 0 | 1 | 2 | 3 | 4 | 5 | 6 | 7 | 8 | 9 | 10 |
| --- | --- | --- | --- | --- | --- | --- | --- | --- | --- | --- |
| Not at all |  | |  |  |  |  |  |  | Very effective | |

1. How much pain do you expect on the pain test?

| 0 | 1 | 2 | 3 | 4 | 5 | 6 | 7 | 8 | 9 | 10 |
| --- | --- | --- | --- | --- | --- | --- | --- | --- | --- | --- |
| No pain |  | |  |  |  |  |  |  | Very  painful | |

Questionnaire 3. No-treatment condition.

1. How comfortable do you feel right now?

| 0 | 1 | 2 | 3 | 4 | 5 | 6 | 7 | 8 | 9 | 10 |
| --- | --- | --- | --- | --- | --- | --- | --- | --- | --- | --- |
| Not at all |  | |  |  |  |  |  |  | Very comfortable | |

1. How is your heart rate right now?

| 0 | 1 | 2 | 3 | 4 | 5 | 6 | 7 | 8 | 9 | 10 |
| --- | --- | --- | --- | --- | --- | --- | --- | --- | --- | --- |
| Completely  normal | | |  |  |  |  |  |  | Very  affected | |

1. How effective do you think a lack of treatment will be for changing your pain sensitivity?

| 0 | 1 | 2 | 3 | 4 | 5 | 6 | 7 | 8 | 9 | 10 |
| --- | --- | --- | --- | --- | --- | --- | --- | --- | --- | --- |
| Not at all |  | |  |  |  |  |  |  | Very effective | |

1. How much pain do you expect on the pain test?

| 0 | 1 | 2 | 3 | 4 | 5 | 6 | 7 | 8 | 9 | 10 |
| --- | --- | --- | --- | --- | --- | --- | --- | --- | --- | --- |
| No pain |  | |  |  |  |  |  |  | Very  painful | |

Supplementary Material 3. Table depicting correlations between measures of pain, anxiety, fear of pain, expectations, and attentional bias. Significant correlations are marked with * (*p* < .05) or ** (*p* < .01).

|  | 1 | 2 | 3 | 4 | 5 | 6 | 7 | 8 | 9 | 10 | 11 | 12 | 13 | 14 | 15 | 16 | 17 |
| --- | --- | --- | --- | --- | --- | --- | --- | --- | --- | --- | --- | --- | --- | --- | --- | --- | --- |
| 1. Pain Thres | - |  |  |  |  |  |  |  |  |  |  |  |  |  |  |  |  |
| 2. Thres Pre-Post | .831** | - |  |  |  |  |  |  |  |  |  |  |  |  |  |  |  |
| 3. Pain Tol | .335 | -.031 | - |  |  |  |  |  |  |  |  |  |  |  |  |  |  |
| 4. Tol Pre-Post | .513** | .477** | .570** | - |  |  |  |  |  |  |  |  |  |  |  |  |  |
| 5. S Anx | .291 | .052 | .337 | -.054 | - |  |  |  |  |  |  |  |  |  |  |  |  |
| 6. S Anx Pre-Post | .209 | .100 | .059 | -.112 | .501** | - |  |  |  |  |  |  |  |  |  |  |  |
| 7. Expect Treat | -.066 | -.091 | -.008 | .017 | -.098 | .100 | - |  |  |  |  |  |  |  |  |  |  |
| 8. Expect Pain | -.041 | -.272 | .334 | .121 | .077 | -.017 | .468** | - |  |  |  |  |  |  |  |  |  |
| 9. T Anx | .042 | -.140 | .006 | -.201 | .256 | -.143 | -.142 | .178 | - |  |  |  |  |  |  |  |  |
| 10. FoP | .012 | .090 | -.117 | .065 | .146 | .044 | -.112 | -.020 | -.102 | - |  |  |  |  |  |  |  |
| 11. FoP – Med | -.126 | -.064 | -.160 | -.019 | -.016 | -.157 | -.084 | -.031 | -.004 | .900** | - |  |  |  |  |  |  |
| 12. FoP – Sev | -.042 | .098 | -.173 | .014 | .209 | .203 | .055 | -.042 | -.250 | .873** | .737** | - |  |  |  |  |  |
| 13. FoP - Min | .169 | .200 | -.012 | .156 | .214 | .115 | -.211 | .010 | -.069 | .898** | .666** | .679** | - |  |  |  |  |
| 14. AB Aff 500 | .348 | .418* | .098 | .301 | -.032 | -.111 | -.158 | .082 | .131 | -.031 | -.151 | .010 | .061 | - |  |  |  |
| 15. AB Sen 500 | .030 | .106 | -.053 | -.064 | .022 | -.050 | .077 | .002 | .121 | .086 | .036 | .117 | .087 | -.031 | - |  |  |
| 16. AB Aff 1250 | -.225 | -.224 | .096 | -.083 | .333 | .066 | -.165 | -.105 | -.071 | .287 | .282 | .452* | .110 | -.242 | .332 | - |  |
| 17. AB Sen 1250 | .276 | .131 | .323 | .332 | .130 | -.068 | -.175 | .138 | .076 | .114 | .076 | .050 | .158 | .363* | -.338 | -.143 | - |

Thres – Threshold

Tol – Tolerance

S Anx – State Anxiety

Expect Treat – Treatment Expectancy

Expect Pain – Pain Expectancy

T Anx – Trait Anxiety

FoP – Fear of Pain

FoP Med – Fear of Pain Medical

FoP Sev – Fear of Pain Severe

FoP Min – Fear of Pain Minor

AB Aff – Attentional Bias Affective

AB Sen – Attentional Bias Sensory
